# Light-Controlled Modulation of the Endocannabinoid System: Photoswitchable Ligands for Cannabinoid and TRPV1 Receptors

**DOI:** 10.3390/ijms27020573

**Published:** 2026-01-06

**Authors:** Alessia Agata Corallo, Carlotta Noli, Antonella Brizzi, Marco Paolino, Claudia Mugnaini, Federico Corelli

**Affiliations:** Dipartimento di Biotecnologie, Chimica e Farmacia, Università degli Studi di Siena, Via A. Moro 2, 53100 Siena, Italy; alessia.corallo2@unisi.it (A.A.C.); carlotta.noli2@unisi.it (C.N.); antonella.brizzi@unisi.it (A.B.); marco.paolino@unisi.it (M.P.); federico.corelli@unisi.it (F.C.)

**Keywords:** photopharmacology, endocannabinoid system (ECS), photoswitchable ligands, cannabinoid receptors ligands, TRPV1 channel ligands, photoisomerization

## Abstract

Photopharmacology is an emerging field in medicinal chemistry that seeks to control the pharmacological effects of target compounds using light. This approach addresses challenges such as limited receptor selectivity by enabling precise spatiotemporal control of therapeutic effects. The light-responsiveness is a central molecular feature used in photopharmacology to modulate the activity of various biological systems, including the endocannabinoid system (ECS). Although the ECS plays a well-established role in the treatment of neurodegeneration, inflammation, and pain, targeting its receptors is challenging due to side effects resulting from receptor activation or inactivation and the incomplete selectivity of available ligands. In this review, we present a comprehensive analysis of the most important ECS photoagonists and photoantagonists, highlighting how this photopharmacological approach overcomes traditional limitations of therapeutic targeting and reduces off-target effects.

## 1. Introduction

Photopharmacology represents an innovative and rapidly expanding field within medicinal chemistry, aimed at the precise and targeted control of pharmacological activity through the use of light as an external stimulus [1,2]. This approach allows for the spatial, temporal, and potentially reversible modulation of therapeutic effects, overcoming certain intrinsic limitations of traditional pharmacology. While systemic drug distribution remains unaffected, selective therapeutic action is achieved through photoactivation within a defined anatomical region [3].

The underlying principle of photopharmacology is the design and modification of bioactive molecules through the incorporation of light-responsive groups capable of altering their configuration or chemical structure in response to light irradiation. Such molecular transformation enables extremely precise control of pharmacological activation both in space and time, significantly reducing the side effects associated with conventional pharmacological treatments and therefore systemic toxicity. Furthermore, it could permit the highly specific targeting of complex and diverse molecular targets, thus promoting the personalization of therapies and adapting interventions to the characteristics of individual patients. Finally, photopharmacology paves the way for the integration of intelligent delivery systems and diagnostic tools based on optical technologies, thereby expanding the available therapeutic and diagnostic potentials [4].

## 2. Photoresponsive Mechanisms

The compounds employed in photopharmacology are mainly classified into five categories according to the mechanism by which light induces structural modification. A distinction is then made among photocleavage control, photodynamic therapy, photothermal control, photodegradation control and, photoconformational control.

**Figure 1:**
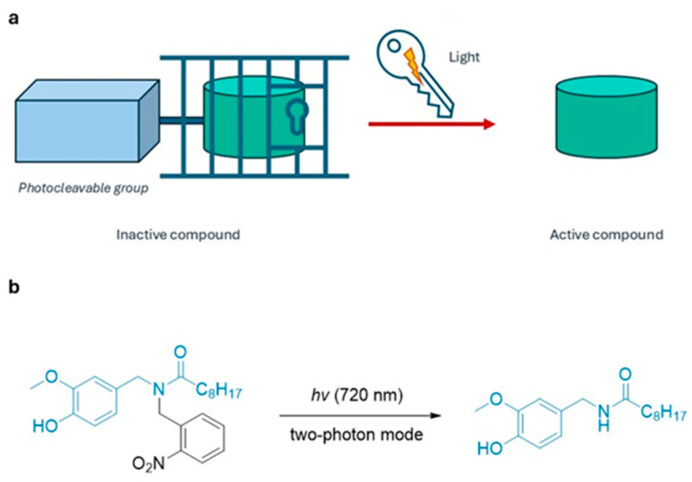
(**a**) Photocleavage control. Drugs are maintained in an inactive form by a photolabile protecting group, which is removed upon irradiation to release the active compound; (**b**) Photorelease of N-vanillylnonanoylamide (VNA) from caged VNAb) [12].

**Photocleavage control.** In this approach molecules remain pharmacologically inactive as long as a suitable protective group remains bound to the drug’s structure (Figure 1a). Exposure to light causes its irreversible removal, releasing the pharmacologically active compound.

The two most studied photocleavable groups in this area are *ortho*-nitrobenzyl and coumarin derivatives [5,6]. The former absorbs in the far ultraviolet (~365 nm) and exhibits limited tissue penetration due to its relatively short wavelength. Coumarin derivatives, on the other hand, have been extensively studied because they have enabled the development of molecules activatable at higher wavelengths, even beyond 500 nm, thus improving compatibility with activation at tissue depths [7]. The limitation of these derivatives lies in their tendency to undergo competing radical pathways during photolysis, which can generate nitroso-aldehydes and other reactive byproducts instead of clean drug release. This side reactivity depends strongly on both the nature of the leaving group and the architecture of the linker connecting the cage to the pharmacophore; accordingly, new o-nitrobenzyl and coumarin-based spacers are being developed to favour heterolytic cleavage, suppress radical channels, and minimize cytotoxic photoproducts [8,9,10,11]. An illustrative example of the biological potential of photoreleasable compounds is provided by Zhao and co-workers, who demonstrated that vanilloid derivatives can be photoreleased in situ to activate TRPV1 receptors on nociceptive neurons, with one-photon quantum efficiency as shown in Figure 1b [12]. Most of the initial examples were cleaved with UV light, which suffers from low tissue permeability and cell damage. Recently, efforts have been made to obtain visible light excitable scaffolds and far-red/near-infrared (FR/NIR) systems operating in the therapeutic window (650–900 nm) [13,14]. These FR/NIR photocages provide more efficient and versatile drug release—especially in hypoxic cancer environments—and show enhanced performance when combined with targeted therapies or functional conjugates.

**Figure 2:**
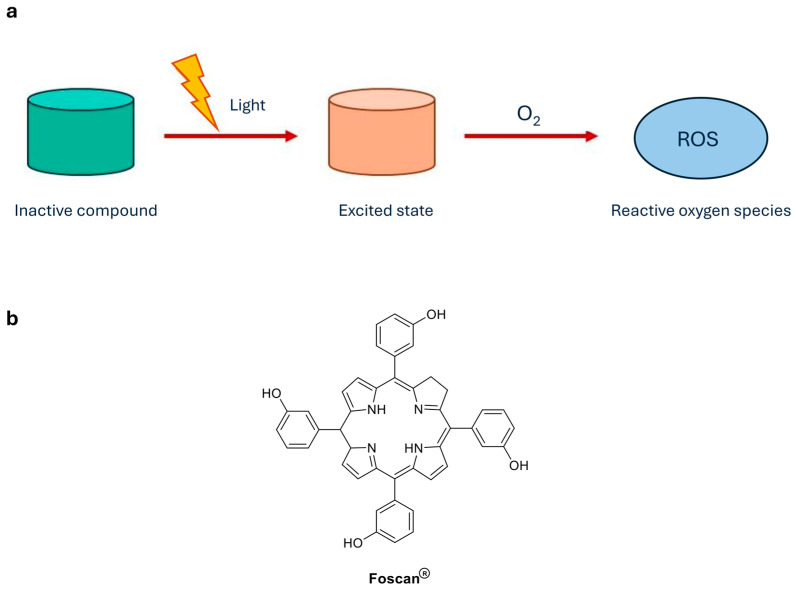
(**a**) Photodynamic therapy. PDT combines a photosensitizer with localized visible light irradiation to generate singlet oxygen and other reactive species that induce confined cell death; (**b**) Chemical structure of the clinically approved agents Foscan^®^ [16].

**Photodynamic therapy (PDT).** It is currently the only clinically approved form of photopharmacology [15]. The treatment involves the administration of a photosensitizing dye, followed by localized irradiation with visible light, which generates an in situ excited state leading to the formation of cytotoxic singlet oxygen or other reactive oxygen species (ROS) that induce localized cell death (Figure 2a).

PDT has been successfully employed in the treatment of cancers, dermatological conditions, and ophthalmic diseases, and has pioneered the therapeutic use of light in combination with pharmacological agents. Clinically approved photosensitizers include Foscan^®^ (depicted in Figure 2b), a chlorin used for the PDT treatment of advanced head and neck, prostate and pancreatic cancers, Photofrin^®^, and other [16,17,18].

Despite its clinical success, PDT still faces several challenges that limit its widespread clinical application:

1. PDT requires effective light delivery to the target site, restricting its therapeutic use to superficial tumours or lesions located in tissues adjacent to accessible organs [19].
2. PDT often relies on high-energy, short-wavelength excitation (UV, blue, or green light), which significantly limits tissue penetration [20].
3. The abnormal vasculature and rapid proliferation of cancer cells in solid tumours lead to hypoxic microenvironments with reduced oxygen availability, which severely compromises the generation of reactive oxygen species and consequently diminishes PDT efficacy.

To address these limitations, chemo-photodynamic therapy (chemo-PDT) has emerged as a complementary strategy, combining conventional chemotherapy with PDT to achieve synergistic anticancer effects [21]. By integrating light-activated cytotoxicity with chemotherapeutic mechanisms that are less dependent on oxygen availability or light penetration, chemo-PDT can partially overcome tumour hypoxia and depth-related constraints while enhancing overall therapeutic efficacy. Recent work has shown that nanoplatform-based chemo-PTD strategies can modulate the hypoxic tumour microenvironment, limiting hypoxia-driven tumour progression [22]. Beyond chemo-PDT, recent evidence indicates that integrating PDT with ferroptosis-inducing mechanisms enhances ROS-driven cytotoxicity and mitigates hypoxia-associated resistance, offering a powerful extension of conventional chemo-photodynamic strategies [23].

**Figure 3:**
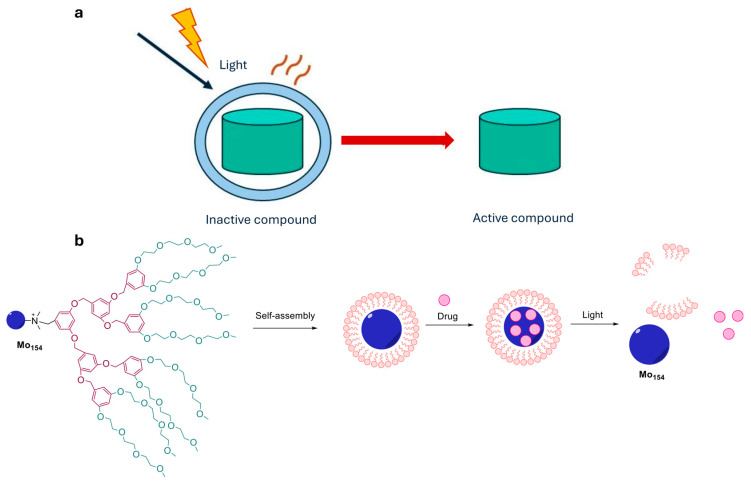
(**a**) Photothermal control. NIR-absorbing nanostructures convert light into localized heat, enabling thermally triggered drug release from temperature-responsive carriers; (**b**) Mo154-based micelles for minimally invasive tumour treatment [25].

**Photothermal control**. Photothermal approaches exploit light energy, typically in the near-infrared (NIR) region, to generate localized heating that can induce thermal ablation or activate controlled drug-release platforms such as gold or gold/silver hybrid nanoparticles (Figure 3a).

These structures can be functionalized with temperature-sensitive polymers or proteins, which release the active compound following conformational changes or phase transitions induced by heat [4,24]. An example of NIR-triggered photothermal-chemotherapy is illustrated by the schematic preparation of Mo154 complex micelles (Figure 3b) [25]. From a therapeutic perspective, photothermal strategies offer several advantages, including deep tissue penetration using NIR light and compatibility with minimally invasive light-delivery techniques. As a result, photothermal control has been widely explored for tumour ablation, combination therapies, and stimulus-responsive drug release in precision medicine [26,27]. Photothermal approaches have already found clinical application primarily as device-based therapies, such as laser interstitial thermal therapy (LITT), which are approved for the treatment of brain tumours and drug-resistant epilepsy [28]. In addition, clinically approved dyes such as indocyanine green have enabled drug-assisted photothermal effects in selected pharmacological settings [29].

**Figure 4:**
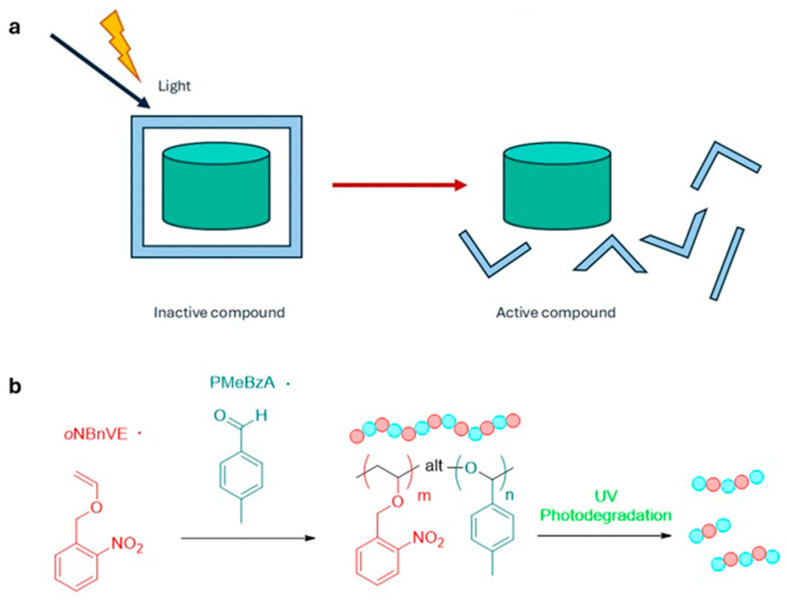
(**a**) Photodegradation control. Polymeric carriers embedding photocleavable units in their backbone undergo light-induced bond scission, fragmentation, or porosity increase, providing on-demand, tunable drug release; (**b**) Design and UV-triggered degradation of an o-nitrobenzyl-functionalized acetal copolymer. An *o*-nitrobenzyl vinyl ether (*o*NBnVE) monomer is copolymerized with *p*-tolualdehyde (*p*MeBzA) to form an alternating acetal backbone bearing photo-deprotectable *o*NBn pendants. UV irradiation induces *o*NBn cleavage, generating hemiacetal units that trigger rapid polymer degradation into low-molecular-weight products [32].

**Photodegradation control**. In this case, polymeric supports or vectors that irreversibly degrade under the action of light, causing controlled local drug release, are used (Figure 4a). These systems exploit photocleavable groups inserted into the polymer or vector structure, which upon irradiation break chemical bonds that maintain material cohesion, causing fragmentation or increased porosity. This mechanism allows targeted and rapid release of entrapped drug molecules with high spatial precision. An additional advantage is the ability to modulate release kinetics by adjusting the intensity and duration of irradiation. However, an important challenge remains the assessment of the biocompatibility and clearance of residual polymer degradation products, which may have systemic effects not yet fully understood [30,31].

As demonstrated by Kubota and Ouchi [32], the incorporation of a photosensitive *ortho*-nitrobenzyl group into vinyl copolymers enables selective backbone degradation via photo-deprotection, without the need for acidic catalysts (Figure 4b).

**Figure 5:**
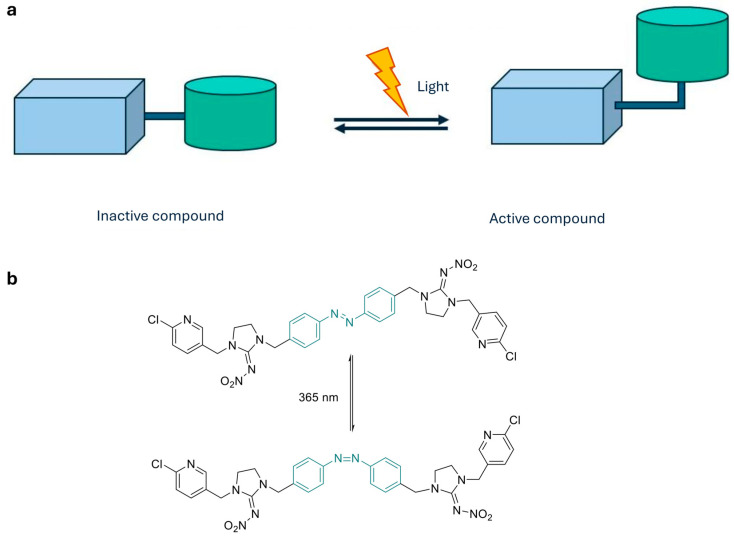
(**a**) Photoconformational control. Photoswitchable ligands incorporate azobenzenes, stilbenes, diarylethenes, or spiropyrans that reversibly isomerize under light, modulating target affinity and activity; (**b**) *Trans*–*cis* geometrical structures and photoisomerization process of AMI-10, an azobenzene-modified imidacloprid derivative developed as a photoswitchable insecticide [33].

**Photoconformational control.** These molecules are able to undergo reversible isomerization between two conformational states (e.g., *cis* and *trans*), modifying their pharmacological activity in response to specific wavelengths (Figure 5a). This type of approach has gained traction in recent years, with the advent of molecular switches such as azobenzene, stilbenes, and diarylethenes [33].

**Figure 6:**
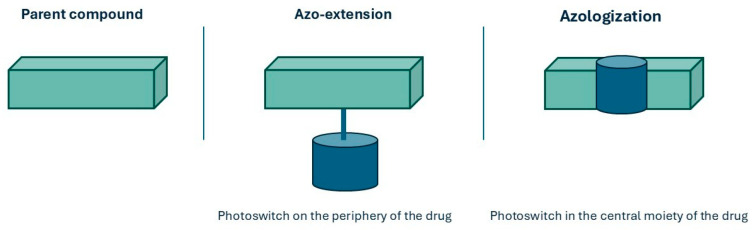
Comparison of photoswitch incorporation strategies in drug design: azo-extension and azologization.

The introduction of azobenzenes can be accomplished by using azo-extension or azo-logization strategies. Azo-extension refers to the strategy of inserting the azoarene photoswitch directly at a strategic position on the parent molecule, extending the conjugation system without significantly altering the original pharmacophore. Azologization instead involves replacing a central structural unit of the parent molecule with an azo moiety, fundamentally changing the molecular scaffold to incorporate the photoswitch (Figure 6) [34].

Following the conformational change induced by light (photoswitch), spontaneous return to the initial conformational state is often observed, generally through a thermally induced process (T type-thermally reversible- photochromic molecules).

**Figure 7:**
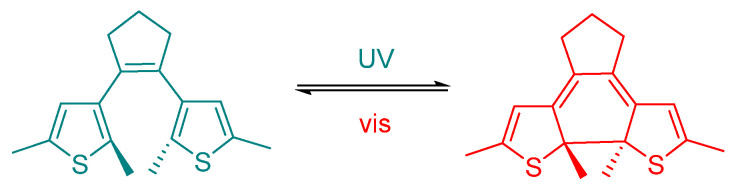
Diarylethenes (DAEs) are robust P-type photochromic switches that reversibly interconvert between open and closed forms under visible light, allowing long-lived control of bioactive photoisomers [42].

This mechanism offers a potential advantage over photocleavage, as molecules activated in the target tissue, once diffused into surrounding areas, can revert to the inactive form, thus reducing the risk of systemic toxicity. Azobenzene derivatives have been extensively studied for their light-triggered isomerization; while regular azobenzene absorbs in the UV, many engineered derivatives show visible-light responsiveness [35]. Although some azobenzene derivatives can undergo metabolic reduction in vivo, thus resulting in rapid elimination or formation of potentially toxic metabolites, their metabolic stability is highly structure-dependent. In fact, many azobenzenes show resistance toward glutathione-mediated reduction and are not substrates for all azoreductases [36]. An example of photoconformational control is provided by azobenzene-modified imidacloprid derivatives, which exhibit light-dependent activity modulation (Figure 5b) [33]. In contrast, systems based on isomerization of carbon–carbon double bond absorb in the ultraviolet region and require more energetic light radiation, which is less effective at penetrating tissues. On the other hand, they exhibit greater chemical stability, being less susceptible to metabolic degradation and, in many cases, safer toxicology [37,38,39,40,41]. In line with stilbene derivatives, diarylethenes (DAEs) are P-type (thermally irreversible, but photochemically reversible) photochromic molecules, endowed with high fatigue resistance and efficient photoconversion between their open and closed forms (Figure 7) [42,43].

Although the structural rearrangement associated with switching is relatively small, DAEs offer exceptional photochemical robustness and can be operated with visible light, making them well suited for applications requiring prolonged retention of a selected photoisomer without continuous irradiation [44].

**Figure 8:**
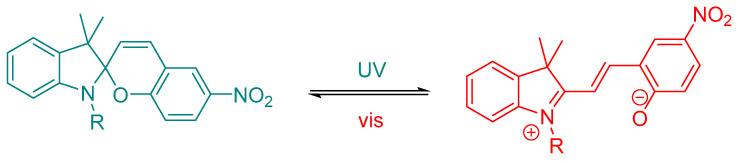
Spiropyrans are photochromic switches that reversibly interconvert between a neutral, non-planar closed spiropyran and ring-opened form, enabling large light-controlled changes in polarity, geometry, and bioactivity [45].

On the other hand, spiropyrans (SPs) function through a light-induced ring-opening mechanism that interconverts a neutral, non-planar spiropyran with a highly polar, conjugated merocyanine form (Figure 8) [45,46].

This transition entails profound changes in polarity, geometry, and electronic distribution, often resulting in marked differences in biological activity between the two isomers.

Together, these photoswitch families exemplify how diverse photochemical scaffolds can expand the scope of photopharmacology, providing complementary properties to azobenzenes and enabling tailored light-responsive behaviour for specific therapeutic or mechanistic applications.

In photopharmacology, reversible photoswitches stand out for their numerous advantages over other light-control strategies of pharmacological activity. Unlike photocleavage and photodegradation, which involve irreversible modifications and the production of potentially toxic chemical residues, photoswitches allow for dynamic and reversible modulation of the therapeutic effect, ensuring precise temporal and spatial control. Compared to photothermal control, photoswitches offer greater chemical compatibility, absence of toxicity from external materials, and high temporal resolution, ideal for modulating rapid or recurring biological events. Furthermore, the possibility of integrating photoswitches into various bioactive molecules without compromising their functionality greatly expands the therapeutic potential of this technology, making it one of the most promising and safest strategies in the field of photopharmacology [47,48].

When discussing reversible photopharmacological tools, several key physicochemical parameters must be considered, as they ultimately determine whether a compound can be effectively applied in biological systems. The most critical among these are activation wavelength, thermal half-life, chemical stability and water-solubility.

Activation wavelength refers to the specific value of light radiation required to induce the transition of the drug from an inactive to an active state. While longer wavelengths (e.g., NIR) offer deeper tissue penetration, shorter wavelengths such as UV are more energetic but have minimal penetration and can induce tissue damage. Since many common photoswitches are activated in the UV range, these limitations must be carefully considered when evaluating their applicability in vivo [49].

Thermal half-life represents the time required for the photoinduced and thermodynamically less stable form of the drug to spontaneously revert to the more stable form. This property is governed by the energy barrier between the two isomers and determines the duration of the drug’s effect in the absence of further light stimulation. An adequate half-life allows effective temporal control of pharmacological activity [50].

Chemical stability of the photoactivatable compound refers to the drug’s resistance to chemical and metabolic degradation, both during storage and within the organism. Good stability ensures that the active principle maintains its efficacy and does not lead to the formation of undesired or potentially harmful products [38].

Water solubility represents a major challenge in photopharmacology, as most photoswitch scaffolds are intrinsically hydrophobic owing to their extended aromatic π-systems. Limited aqueous solubility constitutes not only a pharmacokinetic constraint but also a photophysical and photopharmacological bottleneck, as it can alter the photochemical behaviour of photoswitches by promoting aggregation and affecting photostationary states and switching efficiency under physiological conditions [51]. To overcome these limitations, several design strategies have been explored, including the introduction of polar or ionizable substituents, the use of heteroaromatic or charged photoswitch scaffolds, prodrug or photocaging approaches, and conjugation to hydrophilic carriers. Notably, emerging classes of photoswitches, such as hemiphosphoindigos incorporating phosphinate groups, exemplify innovative solutions for achieving water compatibility while preserving robust photophysical properties [52].

In summary, the efficacy and safety of a light-activatable drug depend on the balanced optimization of these interrelated parameters. A rational and integrated design of photochemical, thermodynamic, and physicochemical properties is therefore essential for the development of precise and reliable light-based therapies [53,54].

Despite significant progress, photopharmacology still presents some critical issues limiting its practical application. Among these, the limited penetration of light in biological tissues, especially for ultraviolet and blue wavelengths, represents a significant obstacle for clinical use at depth. Currently, the clinical applicability of photopharmacology remains largely at the preclinical stage and requires further studies on safety and efficacy [55,56].

Looking ahead, the field of photopharmacology appears extremely promising and continuously evolving. Among the main development lines is the design of photoswitches activatable with light in the red or near-infrared range, which would favour greater tissue penetration and broader clinical applicability [57,58]. On the other hand, the development of new implantable devices for in situ irradiation could represent a turning point for the use of numerous compounds with photomodulable activity [59,60,61].

## 3. Modulation of the Endocannabinoid System Through Light

**Figure 9:**
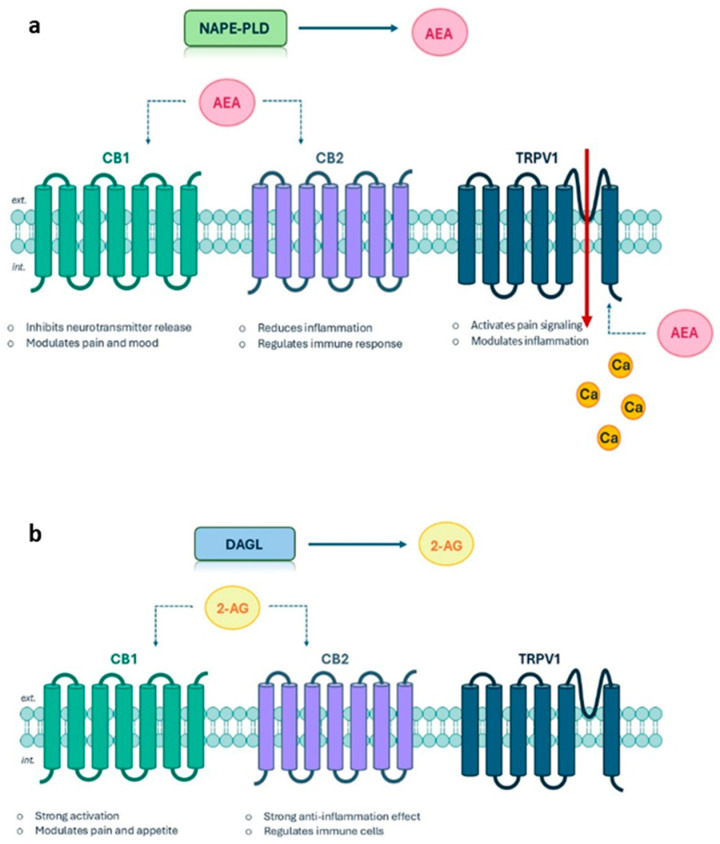
(**a**) Activation of the three ECS receptors through AEA. (**b**) Activation of CB1 and CB2 through 2-AG.

The endocannabinoid system (ECS) is a widespread and complex brain signalling pathway [62,63]. It has a pivotal role in the homeostasis of the organism by influencing physiological and cognitive processes. In fact, ECS regulates pain, humour, appetite, memory, immunity, neuroprotection, and adaptative response of physiological stress [64]. Owing to its multifaceted functions and its capacity to fine-tune neuronal and systemic responses, the ECS represents an attractive and versatile pharmacological target, with potential therapeutic applications in a wide spectrum of pathological states [65]. It is composed of two primary different receptors coupled to the G proteins, i.e., the cannabinoid receptors type-1 (CB1) and type-2 (CB2), and of endogenous cannabinoids such as anandamide (AEA) (Figure 9a) and 2-arachidonoylglycerol (2-AG) (Figure 9b) that, differently to other neurotransmitters, are synthetized ‘on demand’ by N-acyl-transferase (NAPE-PLD) (Figure 9a) and diacylglycerol lipase (DAGL) (Figure 9b), respectively [66]. The CB1 receptor is mainly located in the cerebellum, hippocampus, basal ganglia, and cortex and its activation leads to the inhibition of adenylate cyclase and the influx of calcium. CB2 receptors are primarily located in the peripheral nervous system and the immune system, and their expression is strongly inducible. These receptors exert neuroprotective effects and modulate inflammatory processes by regulating the migration and infiltration of microglia into the brain, thereby reducing the proliferation of free radicals and TNF-α levels.

Disfunctions of the ECS are implicated in the onset and progression of several chronic disorders. Notably, the two receptor subtypes are associated with different pathological profiles: CB1 receptors are predominantly involved in neurological and metabolic conditions such as epilepsy, obesity, schizophrenia, and addiction, while CB2 receptors are mainly linked to inflammatory, oncological, and neurodegenerative diseases including multiple sclerosis and cancer [67].

In addition to the canonical CB1 and CB2 receptors, some endocannabinoids (including anandamide) can also activate the transient receptor potential vanilloid 1 (TRPV1) channel (Figure 9a), a non-selective cation channel implicated in nociception, thermoregulation, and inflammatory responses [68,69]. For this reason, TRPV1 is considered a key component of the ‘extended endocannabinoid system’ [70,71,72]. Its activation by AEA constitutes a major point of integration between these interconnected signalling pathways, whose selective modulation has opened new avenues for therapeutic intervention in pain and inflammation management as well as neuroprotection.

Unfortunately, the development of ligands targeting CB1 [73,74,75,76], CB2 [77,78,79], and TRPV1 [80,81] has faced several challenges. CB1 is abundantly expressed in CNS, where its activation can lead to unwanted psychotropic and cognitive effects, while its blockade has been associated with mood disorders and depression, as observed with inverse agonists such as rimonabant [82]. Conversely, CB2 is mainly expressed in immune and peripheral tissues, but its low basal expression in healthy conditions and the dynamic regulation under pathological states (e.g., inflammation, cancer, neurodegeneration) make it difficult to predict efficacy and safety profiles in clinical settings. Many CB2 agonists that showed potent anti-inflammatory or analgesic effects in animal models failed to translate to humans due to species differences, off-target effects, or poor pharmacokinetic properties [83,84,85]. In the case of TRPV1, which plays a crucial role in nociception, thermoregulation, and inflammation, both agonists and antagonists have displayed significant drawbacks in clinical trials. Potent TRPV1 agonists often cause intense burning sensations and hyperthermia, while antagonists can induce potentially dangerous increases in body temperature, thus narrowing the therapeutic window and limiting clinical translation [86,87].

Moreover, a critical drawback of conventional pharmacological approaches is the lack of spatial and temporal precision: systemically administered ligands act throughout the body, often leading to off-target effects and unwanted systemic responses. For receptors such as CB1, CB2, and TRPV1, widely distributed across peripheral and central compartments, this global activation or inhibition prevents achieving therapeutic efficacy without adverse effects.

To overcome these limitations, strategies that allow precise control over when and where a ligand is active are required. In this context, light-based approaches, such as photopharmacology, offer a unique solution in order to modulate ECS receptors with high spatiotemporal resolution, thereby minimizing systemic side effects and enhancing therapeutic specificity.

## 4. Photopharmacological Approach in the Development of ECS Receptor Ligands

Despite the growing relevance of the ECS as a therapeutic target, conventional drugs acting on its receptors continue to face significant limitations, including poor spatial selectivity and undesirable systemic effects [88]. While endogenous cannabinoids are synthesized and released on demand, in response to specific physiological stimuli [89], conventional synthetic ligands act continuously and indiscriminately, often leading to off-target effects and systemic toxicity. Light-controlled modulation offers a strategy to overcome these limitations by enabling reversible and externally guided regulation of ligand activity, thus minimizing unwanted systemic effects [90].

An example of a photoresponsive strategy that is effective yet conceptually distinct from reversible photopharmacology is provided by ROS-activatable cannabinoid prodrugs developed for combined photodynamic and cannabinoid therapy [91]. 

In this approach, the CB2-targeted cannabinoid mbc94 is covalently linked to a phthalocyanine photosensitizer through a ROS-cleavable linker, such that light irradiation simultaneously induces photodynamic cytotoxicity and irreversible release of the cannabinoid drug, producing potent antitumor effects in CB2R-overexpressing cancer cells. This mechanism, which exemplifies a photoresponsive modality optimized for localized cytotoxic therapy, is therefore poorly suited for reversible, fine-tuned modulation of ECS receptor signalling.

Yin and co-workers reported a CB2-selective agonist masked with a coumarin-based photolabile group, which exhibits markedly reduced receptor affinity in its caged form and undergoes rapid photolysis upon blue-light irradiation to release the active ligand [92]. This approach enables precise spatial and temporal triggering of CB2 activation and is particularly valuable as a chemical tool to study localized receptor populations. However, because ligand activation is irreversible and signalling cannot be dynamically switched off, photocaging strategies are intrinsically limited for fine control of ECS signalling, in contrast to the reversible photoswitch-based approaches discussed below.

Photoconformational control via reversible photoswitches aligns naturally with the requirements of ECS regulation, as it enables bidirectional, spatiotemporally confined switching between active and inactive ligand states without permanent chemical modification. This characteristic underpins the growing emphasis on photoswitchable cannabinoid ligands in the literature and justifies their focus in the subsequent sections.

By integrating a light-responsive moiety into the molecular scaffold, photoswitchable ligands can be toggled between active and inactive states with high temporal resolution and in defined anatomical regions. This approach already applied to several GPCRs targets [93], provides a powerful tool to investigate CB1 and CB2 receptor functions in specific tissues or circuits and represents a promising frontier for precision therapeutics where light-controlled modulation of cannabinoid receptors could maximize efficacy while minimizing systemic and off-target effects [94].

### 4.1. Photoswitchable CB1 Ligands

In the last decade, the photopharmacological approach has also been applied to the design and development of photoresponsive CB1 ligands. By incorporating photoswitchable moieties—primarily azobenzene residues—into known ligand scaffolds, compounds were developed whose pharmacological action can be reversibly controlled by light. This strategy has yielded ligands endowed with both agonist and antagonist functional activity, each with distinct properties that enable fine-tuned manipulation of CB1 signalling and provide a powerful tool for both basic research and potential therapeutic applications.

#### 4.1.1. Photoswitchable CB1 Agonists

Photoswitchable agonists for CB1 receptors were designed by introducing an azobenzene group that, upon irradiation with specific wavelengths, undergoes a conformational change from a more stable isomer to a less stable one. This change in molecular shape alters the ability of the ligand to bind to and activate the receptor, thereby providing optical control over CB1 signalling.

A key example of this approach is the development of photoswitchable Δ^9^-tetrahydrocannabinol (azo-THC) derivatives [95]. These compounds were designed to mimic the binding of the main natural cannabinoid, i.e., THC (**1**). The *trans* and *cis* isomers of these photoswitchable molecules exhibit a different pharmacological profile, allowing for light-dependent modulation of downstream signalling pathways. This optical control has been demonstrated to regulate potassium channel currents and cAMP signalling in living cells, providing a valuable tool for studying the dynamic role of CB1 in neuronal functions. Among the most interesting compounds, there are two analogues of **2**, i.e., derivatives **2** and **3** (Table 1), both obtained using 3-Br-1 as a building block. The structure of the molecules allows light-dependent reversible isomerization between *trans* and *cis* forms through the azobenzene group that confers photoswitchable agonist activity.

Consistently, docking studies showed that both **2** and **3** can bind to the CB1 receptor, with *cis***-2** being more efficacious than *trans***-2**, while the opposite trend was observed for compound **3**.

From a photopharmacological perspective, as shown in Table 1, these compounds demonstrate efficient and reversible photoisomerization at specific wavelengths (around 365 nm for UV-A activation and 450 nm for blue light reversal). Their thermal relaxation times (on the order of hours) facilitate sustained receptor modulation without continuous illumination. This optical control offers significant advantages for investigating CB1 receptor functions in complex biological systems, including neuronal circuits and cell-specific signalling pathways, with minimal off-target effects. Future developments aim to optimize their spectral properties for deeper tissue penetration and broader applicability in vivo.

Recent advances in CB1 photopharmacology have led also to the development of indole-containing photoswitchable compounds. Among these, compound **4** has emerged as a particularly promising candidate due to its high affinity, efficacy, and favourable photophysical properties [96]. The scaffold is characterized by an azobenzene moiety attached at the strategic position of the amide head (**5**). The *cis* isomer exhibits significantly higher affinity and activity compared to the *trans* isomer. The optimized side chain linked to the indole nitrogen (≥5 carbon atoms) enhances hydrophobic interactions within the CB1 binding pocket. As shown in Table 1, upon irradiation with UV light (~365 nm), the target *trans* isomer converts to the *cis* isomer with high efficiency. Furthermore, visible light (~450–550 nm) induces relaxation back to the *trans* form, enabling multiple switching cycles. The *cis* isomer exhibits long-term thermal stability, enabling sustained activation without rapid relaxation. However, over prolonged periods, partial thermal relaxation can still occur, which may compromise temporal precision. The measured fatigue resistance demonstrates minimal degradation over repeated photoisomerization cycles, maintaining consistent switching performance. Finally, visual colour shifts upon isomerization provide an easy readout of the isomeric state. The pharmacological profile of the compound underlines great efficacy of the *cis* isomer, showing an affinity value of approximately 0.18 μM, higher than that of the *trans* isomer (~0.97 μM).

#### 4.1.2. Photoswitchable CB1 Antagonists

The photopharmacological strategy has also been successfully applied to the development of CB1 antagonists. These ligands are designed to block receptor activity in response to light, preventing the action of endogenous or exogenous agonists with high spatiotemporal precision. A prominent example is represented by the work of Rodríguez-Soacha et al. which reports the design and synthesis of analogues of rimonabant [97]. Several derivatives were synthetized incorporating the azoarene photoswitch at a strategic position on the rimonabant scaffold, using azo-extension or azo-logization strategies. As an example, in Table 2 is reported the structure of “photo-rimonabant” **6** achieved via the azo-extension approach.

Like the agonists, also the photoswitchable CB1 antagonists described so far are often “*cis*-ON,” meaning that the *cis* isomer possesses a much higher affinity than the *trans* isomer for the receptor, effectively blocking it.

The azobenzene group allows reversible isomerization between *cis* and *trans* forms upon irradiation with light of specific wavelengths, thereby modulating both affinity and activity at the cannabinoid receptor [97]. Under physiological conditions, the chemical structure of **6** provides high thermal stability to both isomers, which can repeatedly interconvert without significant degradation. Compound **6** exhibited efficient reversible photoisomerization upon irradiation with blue light (~454 nm) and UV light (~366 nm) and achieved high photostationary state (PSS) ratios favouring either isomer depending on the wavelength used, enabling precise control over its receptor affinity. The affinity ratio (*trans*/*cis*) was more than 15-fold in favour of the *cis* photoisomer, which binds CB1 with a *K*_i_ of 29 nM compared to 444 nM for the *trans* form. Interestingly, the *cis* isomer exhibited even stronger affinity for CB1 than the reference compound rimonabant (*K*_i_ = 45 nM). Cell-based assays confirmed that the *cis* isomer acts as a selective antagonist at CB1, effectively inhibiting receptor activity, with negligible activity at CB2. The light-controlled switching allows toggling between active and inactive states, providing dynamic control over receptor modulation. Docking studies revealed that the *cis* isomer interacts favourably within the CB1 binding pocket, engaging key aromatic residues, whereas the *trans* form adopts a conformation with reduced affinity. These interactions underpin the observed differences in binding affinity and activity between isomers. The development of **6** demonstrates the potential of photopharmacology to achieve high-precision modulation of cannabinoid signalling pathways. Its reversible, light-controlled activity offers promising applications in neuroscience research and potential therapeutic interventions targeting the ECS.

### 4.2. Photoswitchable CB2 Ligands

CB2 receptor is a G protein-coupled receptor (GPCR) predominantly expressed in immune cells such as macrophages, lymphocytes, and dendritic cells, as well as in peripheral tissues involved in inflammatory responses. Selective pharmacological modulation of CB2 offers significant advantages by avoiding the psychoactive effects typically associated with CB1 receptor activation in the central nervous system (CNS) (64). Therefore, the development of CB2-specific ligands is considered promising for therapies with an improved safety profile. The identification of photo-responsive ligands for CB2 could overcome traditional limitations by enabling more precise and reversible spatial and temporal control of receptor modulation [98].

#### 4.2.1. Photoswitchable CB2 Agonists

Light-activated agonists targeting the CB2 receptor are molecules that incorporate photosensitive elements capable of reversibly switching between different structural conformations upon exposure to specific wavelengths. A key example of this approach is reported by Sarott and collaborators who developed photoswitchable derivatives of **8**, such as **9** and **10** (Table 3) [99].

These compounds, based on the structure of the CB2 selective agonist HU308 (**8**), carry an azobenzene moiety which allows the optical control of CB2 signalling. The critical alkyl chain at the 3′-position of the resorcinol core was selected as a point of conjugation, employing a late-stage derivatization strategy. In compound **9**, the azobenzene is covalently fused to the resorcinol core, forming a continuous aromatic framework without a phenyl spacer. In contrast, in compound **10** the azobenzene moiety is connected to the resorcinol core, forming an extended conjugated system. With the aim of investigating whether CB2-mediated Ca^2+^ transients could be modulated using light, the azo-HU308 derivatives **9** and **10** were applied to AtT20(CB2) cells preloaded with the calcium indicator Fluo-4. Changes in intracellular calcium levels ([Ca^2+^]_i_) were then monitored before and after photoswitching to assess the optical control exerted by each compound. In the case of **9**, application of the compound in its dark-adapted *trans* configuration produced a pronounced increase in [Ca^2+^]_i_, followed by an approximate 23% reduction upon isomerization with 375 nm irradiation. These results indicate that **9** is more potent in its *trans* configuration. In contrast, **10** did not affect [Ca^2+^]_i_ in its *trans* geometry but determined a significant increase on irradiation at 375 nm, which suggests that only the *cis* configuration has activity at CB2. This complementary photoregulation enables precise and bidirectional control over CB2 signalling in living cells, providing a powerful tool for dissecting the dynamic role of CB2 in cellular excitability and downstream effector pathways [99,102].

The photophysical parameters and potency shift of the CB2 photoswitchable agonists described before are presented in Table 3. In particular, the photosensitive compounds derived from **8**, **9** and **10**, exhibit well-defined photophysical and pharmacological properties. The switching wavelengths employed were 375 nm to induce the *cis* (OFF or ON depending on the compound) geometry and 460 nm to revert to the *trans* (OFF or ON depending on the compound) geometry. Finally, all compounds exhibited good thermal stability in aqueous environments, with *cis* isomer half-lives (t_1_/_2_) of approximately 4–5 h, confirming their biostability and suitability for biological studies [99].

**Figure 10:**
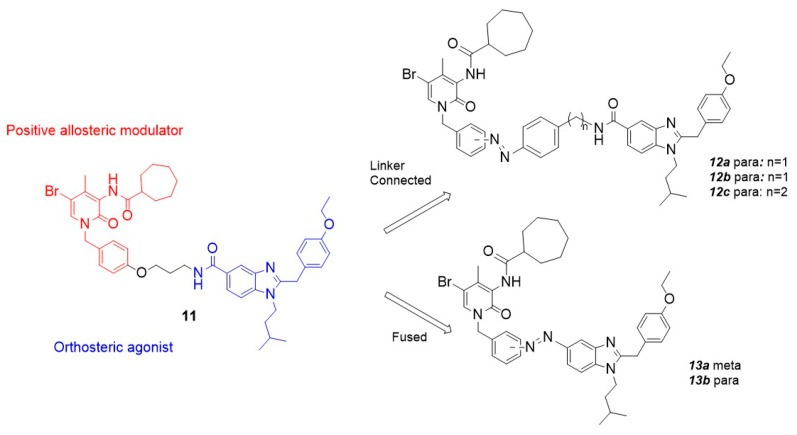
Design of photoswitchable dual-steric ligands based on compound **11** according to two different approaches.

Another noteworthy example of photoswitchable agonists is represented by the dual-steric ligands developed by Steinmüller et al., who designed CB2-photoswitchable compounds based on the previously reported dual-steric ligand **11** (Table 3) [100,103]. To introduce photoreactivity, the original aliphatic linker was replaced with a highly lipophilic azobenzene unit, which had already been successfully employed to generate photoswitchable dual-steric ligands for the muscarinic M1 receptor. Two series of derivatives were developed (Figure 10): in the first, the photoswitchable unit acts as a linker between the orthosteric and allosteric moieties, resulting in an elongated molecular structure (**12a**–**c**); in the second, fused derivatives were synthesized in which the benzimidazole is directly connected to the benzyl ring of the positive allosteric modulator (PAM) via an azo bond (**13a** and **13b**) [100].

The photoisomers of each compound were pharmacologically evaluated for β-arrestin 2 (βarr2)-mediated internalization of CB2R and for their effect on calcium mobilization. While the linker-connected derivatives **12a**–**c**, along with the fused derivative **13a**, exhibited low potency or failed to activate the receptor, the fused derivative **13b** demonstrated a “*cis*-ON” efficacy switch in both assays. The *cis* photoisomer of **13b** (EC_50_ = 824 nM) acted as a full agonist in CB2R internalization and calcium mobilization, showing more than a tenfold increase in potency compared to its *trans* photoisomer. Isomerization to the active *cis* form (ON-isomer) was induced by UV-A light at 375 nm, while reconversion to the *trans* form is triggered by blue light at 530 nm (Table 3). Photostationary ratios indicate high photoisomerization efficiency, with *cis* enrichment reaching approximately 80% under UV irradiation.

A compelling example of photoswitchable ligand design is provided by Steinmüller and collaborators, who developed benzimidazole-based azo-arenes derived from compound **14**, a CB2-selective agonist (Table 3) [101]. The design strategy involved replacing the benzyl group of the parent compound **14** with an azobenzene group in with one of the two typical benzene cores has been replaced by the imidazole moiety of the parent compound. These modifications resulted in substantial changes in the electronic properties of the new molecules. Among these, compound **15** emerged as the lead structure, described as an innovative photoswitch capable of operating under visible light within the 405–520 nm range. Incorporating an azobenzene moiety on the benzimidazole core enabled light-dependent modulation of CB2 activity with a distinctive bias toward β-arrestin2 signalling. Interestingly, compound **15** acts as a “*trans*-ON” efficacy switch, with greater β-arrestin2-mediated receptor internalization and downstream ERK1/2 phosphorylation observed in the *trans* state [103]. From a photophysical perspective, irradiation of **15** with orange light (λ = 590 nm) resulted in an almost quantitative formation of the *trans* isomer (98%), whereas irradiation with purple light (λ = 400 nm) generated a *cis*-enriched photostationary state containing 67% of the cis isomer. Moreover, the compound showed thermal stability, with sufficiently long half-lives (t_1/2_ = 245 min in DMSO and 346 in Tris-buffer) to enable reproducible application in experimental settings [101].

#### 4.2.2. Photoswitchable CB2 Antagonists

Photopharmacology has also proven effective in the design of CB2 antagonists. Typically, such antagonists display a “*cis*-ON” behaviour where the *cis* isomer, binding the receptor with high affinity, effectively blocks its function, while the *trans* isomer shows minimal activity. This reversible photomodulation provides a valuable approach to investigate CB2-mediated physiological roles and disease mechanisms, avoiding the systemic side effects that can arise from conventional pharmacological inhibition [104]. Once again, modifying the parent compound structure by introducing an azobenzene system was successful, resulting in a new family of atypical azobenzene derivatives through the rational remodelling of the CB2 antagonist **16** (Table 4).

Hu and coworkers selected **16** as their starting compound, which represents the only CB2 antagonist with a publicly available X-ray crystal structure in complex with the receptor [96]. This unprecedented yet rational choice allowed them to produce **17** and its analogues using a structure-based drug design approach. In **17**, the azobenzene moiety replaces the adamantyl arm of **16** (subpocket III), representing a targeted remodelling substitution rather than a simple appendage. This strategy is unusual, as adamantane and azobenzene share no obvious structural similarity. However, structure-guided analysis of the receptor pocket revealed that subpocket III contributes approximately 43% of the total binding energy and contains several aromatic residues (Phe872, Phe912, Phe942, Phe183) capable of stabilizing the azobenzene moiety through π–π stacking and hydrophobic interactions. Based on these insights, a library of “azosteres” was developed by retaining the pyrazole core and systematically varying the position and length of the azobenzene linker. Among these analogues, **17** was the most effective, incorporating an ortho-substituted azobenzene with a one-carbon spacer. The outcome was a thermally bistable and highly CB2-selective ligand that demonstrates how careful structure-based pocket analysis, exploiting clusters of hydrophobic and aromatic residues, can be applied to design innovative and selective photopharmacological tools. Data presented in Table 4 highlight *cis*-ON pharmacology for **17**, with the *cis* isomer acting as the active state while the *trans* form is markedly less potent. Although the exact switching wavelengths were not specified, **17** exhibited efficient and reversible *cis–trans* photoisomerization with high thermal biostability, ensuring prolonged stability of the active isomer. Photostationary ratios were reported as high, consistent with efficient light-driven conversion, although precise quantification was not provided. This robust switching behaviour, combined with selective CB2 antagonism, highlights **17** as a valuable photopharmacological probe, showcasing how structure-guided incorporation of azobenzene into atypical scaffolds can generate thermally stable and subtype-selective ligands [98].

### 4.3. Photoswitchable TRPV1 Ligands

While photopharmacology has been extensively applied to the study of classic GPCR cannabinoid receptors (CB1 and CB2), a new frontier of research focuses on the transient receptor potential vanilloid 1 (TRPV1) channel.

#### 4.3.1. Photoswitchable TRPV1 Agonists

The classical exogenous TRPV1 agonist is capsaicin (CAP), the pungent compound found in chilli peppers. By combining the capsaicin (**18**) headgroup with photochromic fatty acids, researchers developed the photoswitchable agonist **19** (Table 5) [105]. In this compound the photochromic tail can reversibly switch between *trans* and *cis* forms under light irradiation, modulating channel activity. In particular, illumination with UV light at 360 nm converts **19** into its *cis* form, which activates TRPV1 at nanomolar concentrations (100–200 nM). In vivo, irradiation of **19** with UV light, induced TRPV1-mediated hyperalgesia, demonstrating that optical control of TRPV1 activity allows reversible and localized modulation of pain signalling. To study the TRPV1 receptor in animal models, an innovative approach that combines photopharmacology and optogenetics has been followed by Frank and co-workers [106]. They utilized **20**, a photoswitchable TRPV1 agonist shown in Table 5, to selectively control neuronal activity. This compound was initially designed and synthetized by Konrad and collaborators [107] by redshifting **19**, cited previously [105]. Konrad showed how these enhanced photoswitches can be directly obtained from already-existing photopharmaceuticals, to allow the use of longer-wavelength light to control protein functions. Due to its properties, **19** is the only example in this review of a photoswitchable compound used in vivo.

This simple and general method enables rapid access to a plethora of redshifted compounds that are better suited for application in living animals and humans. Indeed, **20** was later used as a pioneer by Frank, who, using multifunctional fibres capable of delivering both light and chemical compounds to deep brain regions, overcame the limitations of conventional methods.

Data presented in Table 5 highlight the key characteristics of **20**, confirming its design for complex in vivo functional assays. The ability of this ligand to modulate TRPV1 signalling with light, as documented in this study, opens the door to new investigations into the function of this channel receptor in pain and neuroprotection.

#### 4.3.2. Photoswitchable TRPV1 Antagonists

Photoswitchable antagonists for TRPV1 have been developed based on the structures of the antagonists capsazepine (CPZ-**21**) and BCTC, both containing substituted phenyl rings [108,109]. The authors assumed that the phenyl rings could be replaced by azobenzene moieties and that light-induced *cis–trans* isomerization of these azobenzene units would control the compounds’ antagonistic efficacy.

Among the six derivatives of CPZ, the trifluoromethyl derivative **22** proved to be the most interesting compound (Table 6). Indeed, it acted as a *cis* antagonist of CAP induced TRPV1 currents, whereas it functioned as *trans* antagonist upon voltage activation, demonstrating that a photoswitchable antagonist and an agonist can be combined to achieve optical control of TRPV1 activity.

**Design considerations for photoswitchable ECS ligands.** As illustrated by the examples above, all photoswitchable ligands targeting cannabinoid receptors and TRPV1 channels have been derived from existing lead compounds by incorporating an azobenzene unit as the molecular photoswitch [110]. This derivatization strategy is designed to preserve or even enhance the pharmacological potency of the original ligand while creating a substantial difference in activity between the two photoisomers, thus enabling reliable light-dependent control [110].

Although the number of these photoswitchable ligands is still limited, emerging examples reveal clear structure–activity trends. For CB2 agonists and antagonists, attaching the azobenzene unit via a very short linker (often a single bond) helps maintain receptor affinity while longer or highly flexible spacers tend to reduce potency. The site of attachment on the aromatic ring is equally important: meta-substitution can favour a cis-active (Z-on) ligand because the elongated trans isomer no longer fits into the hydrophobic sub-pocket, whereas para-linked analogues with longer spacers often show diminished activity. These observations align with broader design guidelines for dual-steric GPCR ligands [93], where azobenzenes act as bioisosteric replacements for hydrocarbon chains: the ortho/meta/para substitution pattern and the nature of the linker govern the alignment of the orthosteric and allosteric pharmacophores; overly rigid linkers misalign the pharmacophores, while overly flexible or long linkers reduce selectivity and potency. In TRPV1 agonists that mimic unsaturated lipid chains, the azobenzene is placed within the side chain so that the bent Z isomer can emulate the natural conformation; here again, the E–Z isomerisation influences binding because the linear trans form is less compatible with the binding site. Taken together, these data illustrate that both the connection site and the linker length must be carefully tuned to preserve receptor affinity and achieve effective light-controlled modulation.

## 5. In Vivo Translation of ECS Photopharmacology

Although no published studies have yet demonstrated full in vivo photopharmacological control of the ECS, several closely related GPCR-targeting photoswitches have been successfully applied in transparent or small-animal models under acute experimental conditions.

**Figure 11:**
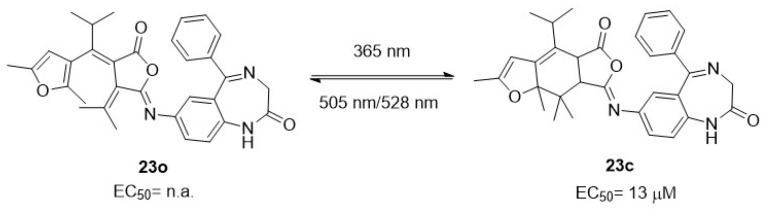
Optical control of GABA_A_R function and behaviour in zebrafish. The fulgimide-based potentiator Fulgazepam is shown in its inactive open isomer **23o** and active closed isomer **23c**, the latter markedly enhancing GABA_A_R currents and increasing larval motility in vivo [111].

For example, the fulgimide-based GABA_A_R potentiator **23**, in its open conformation (**23o**) (Figure 11) [111], does not affect GABA_A_R current amplitude in vitro and does not alter swimming behaviour in zebrafish larvae, a widely used model characterized by optical transparency and well-established drug–behaviour relationships. In contrast, its closed form (**23c**) (Figure 11) strongly potentiates GABA_A_R currents and increases larval motility in a dose-dependent manner, both during prolonged dark periods and under UV illumination.

These applications provide strong proof-of-concept for the feasibility of using photoswitchable ligands in acute in vivo paradigms and support the view that ECS-targeted photopharmaceuticals are well positioned for similar translation. In optically accessible small-animal models such as zebrafish larvae, which have a well-described endocannabinoid system that bears a high similarity to the one in rodents and the human body [112], ECS photopharmacology could be exploited to link receptor activation to rapid behavioural readouts, including locomotion, nociceptive responses, or inflammatory phenotypes, while minimizing systemic and off-target effects that complicate conventional cannabinoid pharmacology [113].

Nevertheless, moving from these early demonstrations to robust in vivo translation may present several challenges:

1. Many cannabinoid photoswitches still rely on UV or blue-light excitation, which severely limits effective control to superficial tissues. In contrast, red- or NIR-addressable photoswitches, capable of deeper tissue penetration with reduced photochemical damage, are only now beginning to be adopted in cannabinoid photopharmacology.
2. Many current ECS photoswitches exhibit suboptimal pharmacokinetic profiles. Poor aqueous solubility and limited bioavailability, combined with a strong tendency to partition non-specifically into lipid-rich tissues, can lead to off-target distribution and systemic effects, ultimately undermining the spatiotemporal selectivity that photopharmacological approaches aim to achieve.
3. Given the high lipophilicity of cannabinoid ligands and the membrane-embedded nature of CB receptors, photoswitch behaviour is highly sensitive to lipid microenvironments, nonspecific protein binding, and metabolic turnover. Such influences can compromise fatigue resistance, shift photostationary equilibria, and alter thermal back-isomerization kinetics, thereby reducing the robustness and reversibility of optical control in vivo.
4. Clinically compatible light-delivery technologies, such as endoscopic, fibre-based, or implantable systems, are needed to safely access deep brain and visceral ECS targets within standard care settings.

Overcoming these limitations will be essential to achieve reliable in vivo control. Ultimately, the success of ECS photopharmacology will depend on its ability to demonstrate clear therapeutic advantages over existing cannabinoid-based strategies in disease-relevant in vivo models.

## 6. Conclusions

In conclusion, functional photopharmacological modulation of cannabinoid receptors, including both CB1 and CB2, has demonstrated significant subtype, pathway, and efficacy selectivity, enabled by the development of several high-fidelity tool compounds. Pioneering photo tools for receptors such as TRPV1 have already facilitated in vivo behavioural studies, opening new avenues for application. Current trends suggest that next-generation probes will expand into more physiological, multiplexed, and therapeutic studies, building on a foundation of precise chemical design integrated with complementary biological and technical approaches. Efforts are ongoing to improve compound stability and selectivity through targeted chemical modifications. Furthermore, integration with miniaturized optical microdevices is underway, allowing non-invasive and more precise applications. An important innovation concerns the possible development of in situ LED implants, positioned subcutaneously, to permit direct irradiation of deep areas, thus overcoming the current limitation of light penetration. Finally, combining these approaches with optical imaging techniques promise real-time monitoring and regulation of photopharmacological therapy, opening new perspectives for personalized and effective treatments. In summary, the convergence of endocannabinsoid pharmacology and photopharmacology offers a unique opportunity to achieve precise, reversible, and localized modulation of CB1 and CB2 receptor activity. By emulating the ECS’s physiological on-demand signalling, photoswitchable ligands can overcome the inherent limitations of conventional cannabinoid drugs, paving the way for innovative therapeutic strategies and advanced research tools.

## Figures and Tables

**Table 1:** Photophysical and pharmacological properties of photoswitchable CB1 agonists.

**Compound**	**Parent Compound**	**Isomerization Type**	***Trans*/*Cis*** **Activity Switching**	**Functional Assay Context**	**Distinctive Elements**
[95]	[95]	Azobenezene meta-linked to a phenyl group replacing the pentyl chain	*Cis*-ON	cAMP, Electrophysiology	Asymmetric synthesis; functional light-dependent switch
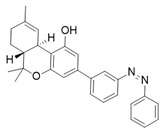	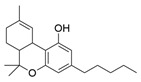	**Switching Wavelengths** *(trans*-to-*cis*/*cis*-to-*trans)*	***E*/*Z* ratio at PSS**	**Thermal** **Stability**	
**2**	**1**	365 nm/450 nm	Exact ratios are not specified	Half-life (t_1/2_) ~216 min in water	
**Compound**	**Parent Compound**	**Isomerization Type**	***Trans*/*Cis* Activity Switching**	**Functional Assay Context**	**Distinctive Elements**
[95]	[95]	Azobenezene replaces the pentyl chain	*Trans*-ON	cAMP, Electrophysiology	Asymmetric synthesis; functional light-dependent switch
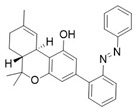	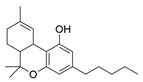	**Switching Wavelengths** *(trans*-to-*cis*/*cis*-to-*trans)*	***E*/*Z* ratio at PSS**	**Thermal** **Stability**	
**3**	**1**	365 nm/450 nm	Exact ratios are not specified	Half-life (t_1/2_) ~216 min in water	
**Compound**	**Parent Compound**	**Isomerization Type**	***Trans/Cis* Activity Switching**	**Functional Assay Context**	**Distinctive Elements**
[96]	[96]	Azobenzene linked at the indole-amide central core	*Cis*-ON (5.4× affinity shift)	Radioligand, arrestin, Ca^2+^	Reversible optical control
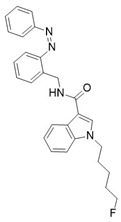	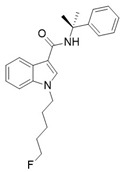	**Switching Wavelengths** *(trans*-to-*cis*/*cis*-to-*trans)*	***E/Z* ratio at PSS**	**Thermal** **Stability**	
**4**	**5**	366 nm/454 nm	PSS_366nm_ = 16/84 PSS_454nm_ = 17/83	Thermal stable in 4:1 DMSO/buffer (pH 7.4) for 3 h at 37 °C. Half-life not reported	

**Table 2:** Photophysical and pharmacological properties of photoswitchable CB1 antagonist **6**.

Compound	Parent Compound	Isomerization Type	*Trans*/*Cis* Activity Switching	Functional Assay Context	Distinctive Elements
[97]	[97]	Azo-extension on position 3	*Cis*-ON (15.3 affinity shift)	Radioligand binding, Ca^2+^ mobilization and cell luminescence assay	First photomodulable CB1 antagonist; high selectivity
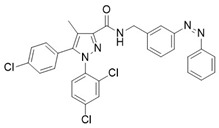	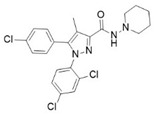	**Switching Wavelengths** *(trans*-to-*cis*/*cis*-to-*trans)*	***E*/*Z* ratio at PSS**	**Thermal** **Stability**	
**6**	Rimonabant **(7)**	366 nm/454 nm	PSS_366nm_ = 4/96 PSS_454nm_ = 74/26	Thermal stable in a period of >3 h in buffer (pH 7.4) at 37 °C	

**Table 3:** Photophysical and pharmacological properties of photoswitchable CB2 agonists.

**Compound**	**Parent Compound**	**Isomerization Type**	***Trans*/*Cis* Activity Switching**	**Functional Assay Context**	**Distinctive Elements**
[99]	[99]	Azobenzene unit fused to the resorcinol core of the parent compound	*Trans*-ON	Real time fluorescent Ca^2+^ imaging in in AtT-20(CB2) cells	Optical control over Ca^2+^ levels in AtT-20(CB2) cells
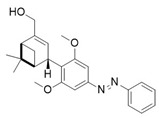	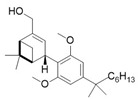	**Switching Wavelengths** *(trans*-to-*cis*/*cis*-to-*trans)*	***E/Z* ratio at PSS**	**Thermal** **Stability**	
**9**	HU-308 (**8**)	365 nm/455 nm	PSS_365nm_ = 20/80 PSS_455nm_ = 83/17	Half-life (t_1/2_) ~2.1 h in water	
**Compound**	**Parent Compound**	**Isomerization Type**	***Trans*/*Cis* Activity Switching**	**Functional Assay Context**	**Distinctive Elements**
[99]	[99]	Azobenzene unit linked to the resorcinol core of the parent compound	*Cis*-ON	Real time fluorescent Ca^2+^ imaging in in AtT-20(CB2) cells	Optical control over Ca^2+^ levels in AtT-20(CB2) cells
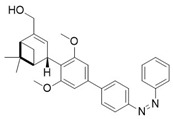	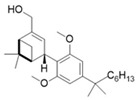	**Switching Wavelengths** *(trans*-to-*cis*/*cis*-to-*trans)*	***E/Z* ratio at PSS**	**Thermal** **Stability**	
**10**	HU-308 (**8**)	365 nm/455 nm	PSS_365nm_ = 51/49 PSS_455nm_ = 87/13	Half-life (t_1/2_) ~1.6 h in water	
**Compound**	**Parent Compound**	**Isomerization Type**	***Trans*/*Cis* Activity Switching**	**Functional Assay Context**	**Distinctive Elements**
[100]	[100]	Azobenzene replaces the original linker being fused on one side to the benzyl group and on the other side with the benzimidazole core	*Cis*-ON, >10× potency shift (17-para)	Internalization, calcium mobilization, and BRET studies	First dual-steric CB2 photoprobe, pathway-specific
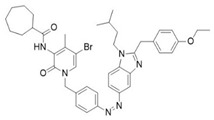	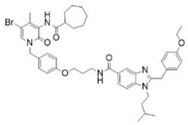	**Switching Wavelengths** *(trans*-to-*cis*/*cis*-to-*trans)*	***E/Z* ratio at PSS**	**Thermal** **Stability**	
**13b**	**11**	365 nm/530 nm	PSS_365nm_ = 5/95 PSS_530nm_ = 85/15	Thermal stable in DMSO for 3 h. Half-life not reported	
**Compound**	**Parent Compound**	**Isomerization Type**	***Trans*/*Cis* Activity Switching**	**Functional Assay Context**	**Distinctive Elements**
[101]	[101]	Azobenzene fused with the imidazole core of the parent compound	*Trans*-ON	β-arrestin2 recruitment and CB2 internalization assays	First pathway-selective CB2 photoprobes
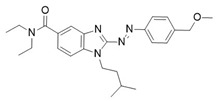	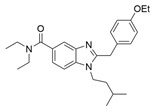	**Switching Wavelengths** *(trans*-to-*cis*/*cis*-to-*trans)*	***E/Z* ratio at PSS**	**Thermal** **Stability**	
**15**	**14**	400 nm/590 nm	PSS_400nm_ = 33/67 PSS_590nm_ = 98/2	Half-life (t_1/2_) ~245 min in DMSO and ~346 min in Tris-buffer (pH 7.4) at room temperature	

**Table 4:** Photophysical and pharmacological properties of photoswitchable CB2 antagonists.

Compound	Parent Compound	Isomerization Type	*Trans*/*Cis* Activity Switching	Functional Assay Context	Distinctive Elements
[98]	[98]	Azobenzene replaces the adamantyl arm	*Cis*-ON	Binding/ Selectivity	Structure-guided remodelling (exploiting pocket clusters)
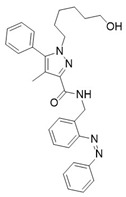	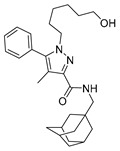	**Switching Wavelengths** *(trans*-to-*cis*/*cis*-to-*trans)*	***E/Z* ratio at PSS**	**Thermal** **Stability**	
**17**	**16**	365 nm/435 nm	PSS_365nm_ = 7/93	Half-life (t_1/2_) ~136 h in DMSO at room temperature	

**Table 5:** Photophysical and pharmacological properties of **19** and **20**.

**Compound**	**Parent Compound**	**Isomerization Type**	***Trans*/*Cis* Activity Switching**	**Functional Assay Context**	**Distinctive Elements**
[105]	[105]	The azobenzene unit replaces the alkyl tail of the capsaicin.	*Cis*-ON	TRPV1-expressing HEK293 cells and primary DRG neurons; ionic currents and Ca^2+^ responses measured under alternating illumination	Fully reversible and reproducible responses
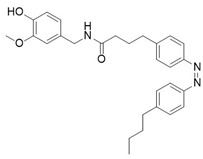	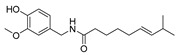	**Switching Wavelengths** *(trans*-to-*cis*/*cis*-to-*trans)*	***E/Z* ratio at PSS**	**Thermal** **Stability**	
**19**	**18**	365 nm/460 nm	No quantitative values reported; description: complete and rapid reversible switching	Half-life (t_1/2_) not reported	
**Compound**	**Parent Compound**	**Isomerization Type**	***Trans*/*Cis* Activity Switching**	**Functional Assay Context**	**Distinctive Elements**
[106]	[106]	Replacement of azobenzene. with a red-shifted azobenzene	*Cis*-ON	In vivo neural/behavioural (VTA mouse)	In vivo activity red-shifted isomerization
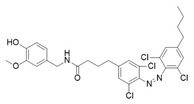	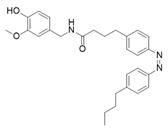	**Switching Wavelengths** *(trans*-to-*cis*/*cis*-to-*trans)*	***E/Z* ratio at PSS**	**Thermal** **Stability**	
**20**	**19**	560 nm/400 nm	No quantitative values reported	Half-life (t_1/2_) not reported	

**Table 6:** Photophysical and pharmacological properties of **22**.

Compound	Parent Compound	Isomerization Type	*Trans*/*Cis* Activity Switching	Functional Assay Context	Distinctive Elements
[108]	[108]	Replacement of chlorobenzene with a substituted azobenzene	*Trans*-ON	Electrophysiology in HEK cells transfected with TRPV1; voltage-dependent assay and Ca^2+^ luminescence assay for CAP response	82% inhibition of CAP-induced TRPV1 currents at 360 nm
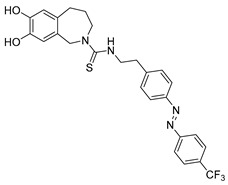	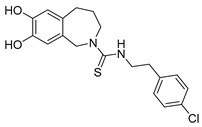	**Switching Wavelengths** *(trans*-to-*cis*/*cis*-to-*trans)*	***E/Z* ratio at PSS**	**Thermal** **Stability**	
**22**	**21**	360 nm/440 nm	No quantitative values reported	Not reported	

## Data Availability

No new data were created or analyzed in this study. Data sharing is not applicable to this article.

## Abbreviations

The following abbreviations are used in this manuscript:

AEA	Anandamide
2-AG	2-Arachidonoylglycerol
cAMP	Cyclic adenosine monophosphate
CAP	Capsaicine
CPZ	Capsazepine
BRET	Bioluminescence resonance energy transfer
CB1	Cannabinoid receptors type-1
CB2	Cannabinoid receptors type-2
CNS	Central nervous system
DAGL	Diacylglycerol lipase
ECS	Endocannabinoid system
ERK1/2	Extracellular signal-regulated kinases 1/2
GPCR	G protein-coupled receptor
IR	Infrared
LED	Light emitting diodes
NAPE–PLD	N-acyl phosphatidylethanolamine phospholipase D
PAM	Positive allosteric modulator
PDT	Photodynamic therapy
PSS	Photostationary state
ROS	Reactive oxygen species
THC	Δ^9^-tetrahydrocannabinol
TNF-α	Tumour necrosis factor alpha
TRPV1	Transient receptor potential vanilloid 1
UV	Ultraviolet
VTA	Ventral tegmental area

## References

[1] Velema W.A., Szymanski W., Feringa B.L. (2014). Photopharmacology: Beyond Proof of Principle. J. Am. Chem. Soc..

[2] Broichhagen J., Frank J.A., Trauner D. (2015). A Roadmap to Success in Photopharmacology. Acc. Chem. Res..

[3] Liu Y., Wang T., Wang W. (2025). Photopharmacology and Photoresponsive Drug Delivery. Chem. Soc. Rev..

[4] Sharma M., Friedman S.H. (2021). The Issue of Tissue: Approaches and Challenges to the Light Control of Drug Activity. ChemPhotoChem.

[5] Li Y., Wang M., Wang F., Lu S., Chen X. (2023). Recent Progress in Studies of Photocages. Smart Mol..

[6] Hansen M.J., Velema W.A., Lerch M.M., Szymanski W., Feringa B.L. (2015). Wavelength-Selective Cleavage of Photoprotecting Groups: Strategies and Applications in Dynamic Systems. Chem. Soc. Rev..

[7] Neto B.A.D., Lapis A.A.M., Mota A.A.R. (2025). Fluorescent coumarin derivatives: Understanding molecular architecture, photophysical, and cell-imaging responses. Targets Heterocycl. Syst..

[8] Holmes C.P. (1997). Model Studies for New o-Nitrobenzyl Photolabile Linkers: Substituent Effects on the Rates of Photochemical Cleavage. J. Org. Chem..

[9] Ma C., Chen Y., Steinmetz M.G. (2006). Photochemical Cleavage and Release of Para-Substituted Phenols from α-Keto Amides. J. Org. Chem..

[10] Johan A.N., Li Y. (2022). Development of Photoremovable Linkers as a Novel Strategy to Improve the Pharmacokinetics of Drug Conjugates and Their Potential Application in Antibody–Drug Conjugates for Cancer Therapy. Pharmaceuticals.

[11] Klán P., Šolomek T., Bochet C.G., Blanc A., Givens R., Rubina M., Popik V., Kostikov A., Wirz J. (2013). Photoremovable Protecting Groups in Chemistry and Biology: Reaction Mechanisms and Efficacy. Chem. Rev..

[12] Zhao J., Sankaranarayanan A., Paik B.H., Kim J., Shim H., Kao J.P.Y. (2006). Caged Vanilloid Ligands for Activation of TRPV1 Receptors by 1- and 2-Photon Excitation. Biochemistry.

[13] Josa-Culleré L., Llebaria A. (2021). In the Search for Photocages Cleavable with Visible Light: An Overview of Recent Advances and Chemical Strategies. ChemPhotoChem.

[14] Xiong H., Xu Y., Kim B., Rha H., Zhang B., Li M., Yang G.F., Kim J.S. (2023). Photo-Controllable Biochemistry: Exploiting the Photocages in Phototherapeutic Window. Chem.

[15] Aebisher D., Czech S., Dynarowicz K., Misiołek M., Komosińska-Vassev K., Kawczyk-Krupka A., Bartusik-Aebisher D. (2024). Photodynamic Therapy: Past, Current, and Future. Int. J. Mol. Sci..

[16] Glowacka-Sobotta A., Czarczynska-Goslinska B., Ziental D., Wysocki M., Michalak M., Güzel E., Sobotta L. (2024). Versatile Porphyrin Arrangements for Photodynamic Therapy—A Review. Nanomaterials.

[17] Wiehe A., Senge M.O. (2023). The Photosensitizer Temoporfin (mTHPC)—Chemical, Pre-Clinical and Clinical Developments in the Last Decade. Photochem. Photobiol..

[18] Wang X., Peng J., Meng C., Feng F. (2024). Recent Advances for Enhanced Photodynamic Therapy: From New Mechanisms to Innovative Strategies. Chem. Sci..

[19] Zhou Z., Song J., Nie L., Chen X. (2016). Reactive Oxygen Species Generating Systems Meeting Challenges of Photodynamic Cancer Therapy. Chem. Soc. Rev..

[20] Fan W., Huang P., Chen X. (2016). Overcoming the Achilles’ heel of photodynamic therapy. Chem. Soc. Rev..

[21] Xie X., Sun T., Pan H., Ji D., Xu Z., Gao G., Miao J., Wang L., Zhang Y., Liu J. (2024). Development of Novel β-Carboline/Furylmalononitrile Hybrids as Type I/II Photosensitizers with Chemo-Photodynamic Therapy and Minimal Toxicity. Mol. Pharm..

[22] Shen W., Han G., Yu L., Yang S., Li X., Zhang W., Pei P. (2022). Combined Prussian Blue Nanozyme Carriers Improve Photodynamic Therapy and Effective Interruption of Tumor Metastasis. Int. J. Nanomed..

[23] Zou Y., Chen J., Luo X., Qu Y., Zhou M., Xia R., Wang W., Zheng X. (2024). Porphyrinengineered nanoscale metal-organic frameworks: Enhancing photodynamic therapy and ferroptosis in oncology. Front. Pharmacol..

[24] Yavuz M.S., Cheng Y., Chen J., Cobley C.M., Zhang Q., Rycenga M., Xie J., Kim C., Song K.H., Schwartz A.G. (2009). Gold Nanocages Covered by Smart Polymers for Controlled Release with Near-Infrared Light. Nat. Mater..

[25] Kong X., Zhang X., Wang Y., Zhang B. (2025). Recent Advances of Photothermal Materials for Biomedical Applications. ACS Omega.

[26] Li X., Lovell J.F., Yoon J., Chen X. (2020). Clinical development and potential of photothermal and photodynamic therapies for cancer. Nat. Rev. Clin. Oncol..

[27] Chen G., Zhao Y., Xu Y., Zhu C., Liu T., Wang K. (2020). Chitosan nanoparticles for oral photothermally enhanced photodynamic therapy of colon cancer. Int. J. Pharm..

[28] Atel B., Kim A.H. (2020). Laser Interstitial Thermal Therapy. Mo. Med..

[29] Shirata C., Kaneko J., Inagaki Y., Kokudo T., Sato M., Kiritani S., Akamatsu N., Arita J., Sakamoto Y., Hasegawa K. (2017). Near-infrared photothermal/photodynamic therapy with indocyanine green induces apoptosis of hepatocellular carcinoma cells through oxidative stress. Sci. Rep..

[30] Fomina N., McFearin C., Sermsakdi M., Edigin O., Almutairi A. (2010). UV and Near-IR Triggered Release from Polymeric Nanoparticles. J. Am. Chem. Soc..

[31] Men Y., Brevé T.G., Liu H., Denkova A.G., Eelkema R. (2021). Photocleavable Thioacetal Block Copolymers for Controlled Release. Polym. Chem..

[32] Kubota H., Ouchi M. (2023). Rapid and Selective Photo-Degradation of Polymers: Design of an Alternating Copolymer with an o-Nitrobenzyl Ether Pendant. Angew. Chem. Int. Ed..

[33] Xu Z., Shi L., Jiang D., Cheng J., Shao X., Li Z. (2015). Azobenzene-Modified Imidacloprid Derivatives as Photoswitchable Insecticides: Steering Molecular Activity in a Controllable Manner. Sci. Rep..

[34] Cacciarini M., Woolley G.A., Szymanski W., Simeth N.A. (2025). Introduction to photoswitches and photopharmacology. Org. Biomol. Chem..

[35] Jerca F.A., Jerca V.V., Hoogenboom R. (2022). Advances and opportunities in the exciting world of azobenzenes. Nat. Rev. Chem..

[36] Zhou X., Du L., Li M. (2025). Recent Progress in Azobenzene-Based In Vivo Photopharmacology. Med. Res. Rev..

[37] Lerch M.M., Hansen M.J., van Dam G.M., Szymanski W., Feringa B.L. (2016). Emerging Targets in Photopharmacology. Angew. Chem. Int. Ed..

[38] SeethaLekshmi S., Thakur T.S., Varughese S. (2021). Photoinstability in Active Pharmaceutical Ingredients: Crystal Engineering as a Mitigating Measure. J. Photochem. Photobiol. C Photochem. Rev..

[39] Poggialini F., Governa P., Vagaggini C., Maramai S., Lamponi S., Mugnaini C., Brizzi A., Purgatorio R., de Candia M., Catto M. (2025). Light-Mediated Activation/Deactivation Control and In Vitro ADME–Tox Profiling of a Donepezil-like Dual AChE/MAO-B Inhibitor. Eur. J. Pharm. Sci..

[40] Paolino M., De Candia M., Purgatorio R., Catto M., Saletti M., Tondo A.R., Nixolotti O., Cappelli A., Brizzi A., Mugnaini C. (2023). Investigation on Novel *E/Z* 2-Benzylideneindan-1-One-Based Photoswitches with AChE and MAO-B Dual Inhibitory Activity. Molecules.

[41] Paolino M., Rullo M., Maramai S., de Candia M., Pisani L., Catto M., Mugnaini C., Brizzi A., Cappelli A., Olivucci M. (2022). Design, synthesis and biological evaluation of light-driven on–off multitarget AChE and MAO-B inhibitors. RSC Med. Chem..

[42] Irie M., Fukaminato T., Matsuda K., Kobatake S. (2014). Photochromism of Diarylethene Molecules and Crystals: Memories, Switches, and Actuators. Chem. Rev..

[43] Zhang J., Tian H. (2018). The Endeavor of Diarylethenes: New Structures, High Performance, and Bright Future. Adv. Opt. Mater..

[44] Guo H., Dai J., Deng L., Zhang Z., Tian H., Zhang J. (2025). Photopharmacology beyond azobenzene photoswitches. Responsive Mater..

[45] Kortekaas L., Browne W.R. (2019). The evolution of spiropyran: Fundamentals and progress of an extraordinarily versatile photochrome. Chem. Soc. Rev..

[46] Rad J.K., Balzade Z., Mahdavian A.R. (2022). Spiropyran-based advanced photoswitchable materials: A fascinating pathway to the future stimuli-responsive devices. J. Photochem. Photobiol. C Photochem. Rev..

[47] Fuchter M.J. (2020). On the promise of photopharmacology using photoswitches: A medicinal chemist’s perspective. J. Med. Chem..

[48] Arkhipova V., Fu H., Hoorens M.W.H., Trinco G., Lameijer L.N., Marin E., Ben L., Feringa B.L., Poelarends G.J., Szymanski W. (2021). Structural Aspects of Photopharmacology: Insight into the Binding of Photoswitchable and Photocaged Inhibitors to the Glutamate Transporter Homologue. J. Am. Chem. Soc..

[49] Rapp T.L., DeForest C.A. (2021). Targeting drug delivery with light: A highly focused approach. Adv. Drug Deliv. Rev..

[50] Axelrod S., Shakhnovich E., Gómez-Bombarelli R. (2023). Thermal Half-Lives of Azobenzene Derivatives: Virtual Screening Based on Intersystem Crossing Using a Machine Learning Potential. ACS Cent. Sci..

[51] Volarić J., Szymanski W., Feringa B.L., Velema W.A. (2021). Molecular Photoswitches in Aqueous Environments. Chem. Soc. Rev..

[52] Kohl F., Vogl T., Hampel F., Dube H. (2025). Hemiphosphoindigos as a Platform for Chiroptical or Water Soluble Photoswitching. Nat. Commun..

[53] Weinstain R., Slanina T., Kand D., Klán P. (2020). Visible-to-NIR-Light Activated Release: From Small Molecules to Nanomaterials. Chem. Rev..

[54] Szymański W., Beierle J.M., Kistemaker H.A.V., Velema W.A., Feringa B.L. (2013). Reversible photocontrol of biological systems by the incorporation of molecular photoswitches. Chem. Rev..

[55] Ankenbruck N., Courtney T., Naro Y., Deiters A. (2018). Optochemical Control of Biological Processes in Cells and Animals. Angew. Chem. Int. Ed..

[56] Yang Y., Long K., Chu Y., Lu H., Wang W., Zhan C. (2024). Photoresponsive Drug Delivery Systems: Challenges and Progress. Adv. Funct. Mater..

[57] Metuh P., Petersen P.M., Ou Y. (2024). Recent Advances in Wireless Optoelectronic Biomedical Implants. Laser Photonics Rev..

[58] Guesdon-Vennerie A., Couvreur P., Ali F., Pouzoulet F., Roulin C., Martínez-Rovira I., Bernadat G., Legrand F.X., Bourgaux C., Mazars C.L. (2022). Breaking photoswitch activation depth limit using ionising radiation stimuli adapted to clinical application. Nat. Commun..

[59] Wang C., Yu Q., Zhang X., Wu M.X., Lu M. (2025). Flexible, implantable, and wearable LED devices based on the perspective of photomedicine: Progress and potential medical applications. Mater. Today.

[60] Qazi R., Kim C.Y., Kang I., Binazarov D., McCall J.G., Jeong J.W. (2021). Implantable Optofluidic Systems for Wireless In Vivo Photopharmacology. ChemPhotoChem.

[61] Wang C., Yu Q., Li M., Chen H., Fan H., Ma Y., Zhang Z., Wu M.X., Lu M. (2025). Challenges and opportunities in next-generation LED therapeutic devices. Light Sci. Appl..

[62] Piomelli D. (2003). The molecular logic of endocannabinoid signalling. Nat. Rev. Neurosci..

[63] Lu H.C., Mackie K. (2021). Review of the Endocannabinoid System. Biol. Psychiatry Cogn. Neurosci. Neuroimaging.

[64] Mechoulam R., Parker L.A. (2013). The endocannabinoid system and the brain. Annu. Rev. Psychol..

[65] Di Marzo V., Bifulco M., De Petrocellis L. (2004). The endocannabinoid system and its therapeutic exploitation. Nat. Rev. Drug Discov..

[66] Martin J.B. (1999). Molecular Basis of the Neurodegenerative Disorders. N. Engl. J. Med..

[67] Grossi E., Mancini A., Buscema M., Savarè R., Intraligi M. (2006). SAAB—Sistemi Artificiali Adattivi in Biomedicina: Donepezil e disturbi comportamentali in pazienti con sindrome di Alzheimer: Profilo prototipico dei Responders e Non-responders attraverso un nuovo modello di Reti Neurali Artificiali. Sist. Artif. Adattivi Biomed..

[68] Caterina M.J., Schumacher M.A., Tominaga M., Rosen T.A., Levine J.D., Julius D. (1997). The capsaicin receptor: A heat-activated ion channel in the pain pathway. Nature.

[69] Romeo I., Brizzi A., Pessina F., Ambrosio F.A., Aiello F., Belardo C., Carullo G., Costa G., De Petrocellis L., Frosini M. (2023). In Silico-Guided Rational Drug Design and Synthesis of Novel 4-(Thiophen-2-yl)butanamides as Potent and Selective TRPV1 Agonists. J. Med. Chem..

[70] Di Marzo V. (2009). The endocannabinoid system: Its general strategy of action, tools for its pharmacological manipulation and potential therapeutic exploitation. Pharmacol. Res..

[71] Lowe H., Toyang N., Steele B., Bryant J., Ngwa W. (2021). The Endocannabinoid System: A Potential Target for the Treatment of Various Diseases. Int. J. Mol. Sci..

[72] Xiao T., Sun M., Zhao C., Kang J. (2023). TRPV1: A promising therapeutic target for skin aging and inflammatory skin diseases. Front. Pharmacol..

[73] Rinaldi-Carmona M., Barth F., Héaulme M., Shire D., Calandra B., Congy C., Martinez S., Maruani J., Néliat G., Caput D. (1994). SR141716A, a potent and selective antagonist of the brain cannabinoid receptor. FEBS Lett..

[74] Lan R., Lu Q., Fan P., Gatley J., Volkow N.D., Fernando S.R., Volkow N.D., Pertwee R., Makriyannis A. (1999). Design and synthesis of the CB1 selective cannabinoid antagonist AM281: A potential human SPECT ligand. AAPS PharmSci.

[75] Brizzi A., Brizzi V., Cascio M.G., Bisogno T., Sirianni R., Di Marzo V. (2005). Design, Synthesis, and Binding Studies of New Potent Ligands of Cannabinoid Receptors. J. Med. Chem..

[76] Brizzi A., Cascio M.G., Brizzi V., Bisogno T., Dinatolo M.T., Martinelli A., Tuccinardi T., Di Marzo V. (2007). Design, synthesis, binding, and molecular modeling studies of new potent ligands of cannabinoid receptors. Bioorg. Med. Chem..

[77] Mugnaini C., Brizzi A., Ligresti A., Allarà M., Lamponi S., Vacondio F., Silva C., Mor M., Di Marzo V., Corelli F. (2016). Investigations on the 4-Quinolone-3-carboxylic Acid Motif. 7. Synthesis and Pharmacological Evaluation of 4-Quinolone-3-carboxamides and 4-Hydroxy-2-quinolone-3-carboxamides as High Affinity Cannabinoid Receptor 2 (CB2R) Ligands with Improved Aqueous Solubility. J. Med. Chem..

[78] Ibrahim M.M., Porreca F., Lai J., Albrecht P.J., Rice F.L., Khodorova A., Davar G., Makriyannis A., Vanderah T.W., Mata H.P. (2005). CB2 cannabinoid receptor activation produces antinociception by stimulating peripheral release of endogenous opioids. Proc. Natl. Acad. Sci. USA.

[79] Hanuš L., Breuer A., Tchilibon S., Shiloah S., Goldenberg D., Horowitz M., Pertwee R.G., Ross R.A., Mechoulam R., Fride E. (1999). HU-308: A specific agonist for CB2, a peripheral cannabinoid receptor. Proc. Natl. Acad. Sci. USA.

[80] Brizzi A., Maramai S., Aiello F., Baratto M.C., Corelli F., Mugnaini C., Paolino M., Scorzelli F., Aldinucci C., De Petrocellis L. (2022). Lipoic/Capsaicin-Related Amides: Synthesis and Biological Characterization of New TRPV1 Agonists Endowed with Protective Properties against Oxidative Stress. Int. J. Mol. Sci..

[81] Maramai S., Mugnaini C., Paolino M., Schiano Moriello A., De Petrocellis L., Corelli F., Aiello F., Brizzi A. (2025). Indole-2-Carboxamide as an Effective Scaffold for the Design of New TRPV1 Agonists. Molecules.

[82] Bosquez-Berger T., Szanda G.Ó., Straiker A. (2023). Requiem for Rimonabant: Therapeutic Potential for Cannabinoid CB1 Receptor Antagonists after the Fall. Drugs Drug Candidates.

[83] Carruthers E.R., Grimsey N.L. (2024). Cannabinoid CB2 receptor orthologues; in vitro function and perspectives for preclinical to clinical translation. Br. J. Pharmacol..

[84] Naikoo R.A., Painuli R., Akhter Z., Singh P.P. (2024). Cannabinoid receptor 2 (CB2) modulators: A patent review (2016–2024). Bioorg. Chem..

[85] Guenther K.G., Wirt J.L., Oliva I., Saberi S.A., Crystal J.D., Hohmann A.G. (2025). The cannabinoid CB2 agonist LY2828360 suppresses neuropathic pain behavior and attenuates morphine tolerance and conditioned place preference in rats. Neuropharmacology.

[86] Laklouk M., Baranidharan G. (2016). Profile of the capsaicin 8% patch for the management of neuropathic pain associated with postherpetic neuralgia: Safety, efficacy, and patient acceptability. Patient Prefer. Adherence.

[87] Koivisto A.P., Belvisi M.G., Gaudet R., Szallasi A. (2021). Advances in TRP channel drug discovery: From target validation to clinical studies. Nat. Rev. Drug Discov..

[88] Di Marzo V. (2018). New approaches and challenges to targeting the endocannabinoid system. Nat. Rev. Drug Discov..

[89] Alger B.E., Kim J. (2011). Supply and demand for endocannabinoids. Trends Neurosci..

[90] Ricart-Ortega M., Font J., Llebaria A. (2019). GPCR photopharmacology. Mol. Cell. Endocrinol..

[91] Ling X., Zhang S., Liu Y., Bai M. (2018). Light-activatable cannabinoid prodrug for combined and target-specific photodynamic and cannabinoid therapy. J. Biomed. Opt..

[92] Yin J., Sharma R., Tyndall J.D.A., Grimsey N.-L., Vernall A.J. (2023). Synthesis and Characterization of a Cannabinoid Type 2 Receptor Photoactivated Prodrug. ChemPhotoChem.

[93] Mori S., Arella D., Decker M. (2025). Photoswitchable allosteric and dualsteric ligands in GPCR pharmacology. Trends Pharmacol. Sci..

[94] Basagni F., Rosini M., Decker M. (2020). Functionalized Cannabinoid Subtype 2 Receptor Ligands: Fluorescent, PET, Photochromic and Covalent Molecular Probes. ChemMedChem.

[95] Westphal M.V., Schafroth M.A., Sarott R.C., Imhof M.A., Bold C.P., Leippe P., Amey Dhopeshwarkar A., Grandner J.G., Katritch V., Mackie K. (2017). Synthesis of Photoswitchable 9-Tetrahydrocannabinol Derivatives Enables Optical Control of Cannabinoid Receptor 1 Signaling. J. Am. Chem. Soc..

[96] Rodríguez-Soacha D.A., Steinmüller S.A.M., Işbilir A., Fender J., Deventer M.H., Ramírez Y.A., Tutov A., Sotriffer C., Stove C.P., Lorenz K. (2022). Development of an Indole-Amide-Based Photoswitchable Cannabinoid Receptor Subtype 1 (CB1R) “Cis-On” Agonist. ACS Chem. Neurosci..

[97] Rodríguez-Soacha D.A., Fender J., Ramírez Y.A., Collado J.A., Muñoz E., Maitra R., Sotriffer C., Lorenz K., Decker M. (2021). “Photo-Rimonabant”: Synthesis and Biological Evaluation of Novel Photoswitchable Molecules Derived from Rimonabant Lead to a Highly Selective and Nanomolar “Cis-On” CB1R Antagonist. ACS Chem. Neurosci..

[98] Hu T., Zheng G., Xue D., Zhao S., Li F., Zhou F., Zhao F., Xie L., Tian C., Hua T. (2021). Rational Remodeling of Atypical Scaffolds for the Design of Photoswitchable Cannabinoid Receptor Tools. J. Med. Chem..

[99] Sarott R.C., Viray A.E.G., Pfaff P., Sadybekov A., Rajic G., Katritch V., Carreira E.M., Frank J.A. (2021). Optical Control of Cannabinoid Receptor 2-Mediated Ca^2+^ Release Enabled by Synthesis of Photoswitchable Probes. J. Am. Chem. Soc..

[100] Steinmüller S.A.M., Tutov A., Hislop J.N., Decker M. (2023). Bridging the Binding Sites 2.0: Photoswitchable Dualsteric Ligands for the Cannabinoid 2 Receptor. ACS Chem. Neurosci..

[101] Steinmüller S.A.M., Fender J., Deventer M.H., Tutov A., Lorenz K., Stove C.P., Hislop J.N., Decker M. (2023). Visible-Light Photoswitchable Benzimidazole Azo-Arenes as β-Arrestin2-Biased Selective Cannabinoid 2 Receptor Agonists. Angew. Chem. Int. Ed..

[102] Viray A.E.G., Frank J.A. (2025). The photoswitchable cannabinoid azo-HU308 enablesoptical control of Ca^2+^ dynamics in INS-1 b-cells viaoff-target effects on TRPC channels. FEBS Open Bio.

[103] Tutov A., Steinmüller S.A.M., Ramírez Y.A., Jack C.E., Rodríguez-Soacha D.A., Sotriffer C., Decker M. (2023). Bridging the Binding Sites: Dualsteric Ligands for the Cannabinoid 2 Receptor (CB2R). Adv. Ther..

[104] Kobauri P., Dekker F.J., Szymanski W., Feringa B.L. (2023). Rational Design in Photopharmacology with Molecular Photoswitches. Angew. Chem. Int. Ed..

[105] Frank J.A., Moroni M., Moshourab R., Sumser M., Lewin G.R., Trauner D. (2015). Photoswitchable fatty acids enable optical control of TRPV1. Nat. Commun..

[106] Frank J.A., Antonini M.J., Chiang P.H., Canales A., Konrad D.B., Garwood I.C., Rajic G., Koehler F., Fink Y., Anikeeva P. (2020). In Vivo Photopharmacology Enabled by Multifunctional Fibers. ACS Chem. Neurosci..

[107] Konrad D.B., Frank J.A., Trauner D. (2016). Synthesis of Redshifted Azobenzene Photoswitches by Late-Stage Functionalization. Chem. Eur. J..

[108] Stein M., Breit A., Fehrentz T., Gudermann T., Trauner D. (2013). Optical control of TRPV1 channels. Angew. Chem. Int. Ed..

[109] Gataulina E.D., Nikolaev M.V., Tikhonov D.B. (2024). Design of Soluble Compounds for Optical Control of Tetrameric P-Loop Ion Channels. J. Evol. Biochem. Physiol..

[110] Wermuth C.G. (2006). Similarity in drugs: Reflections on analogue design. Drug Discov. Today.

[111] Rustler K., Maleeva G., Gomila A.M.J., Gorostiza P., Bregestovski P., Kçnig B. (2020). Optical Control of GABA A Receptors with a Fulgimide-Based Potentiator. Chem. Eur. J..

[112] Oltrabella F., Melgoza A., Nguyen B., Guo S. (2017). Role of the endocannabinoid system in vertebrates: Emphasis on the zebrafish model. Develop. Growth Differ..

[113] Campolongo P., Trezza V. (2012). The endocannabinoid system: A key modulator of emotions and cognition. Front. Behav. Neurosci..

